# The Role of Motion Correction Tools in Left Ventricular Functional Parameters Measured by Gated [^13^N]NH_3_ PET/CT

**DOI:** 10.3390/diagnostics16091377

**Published:** 2026-05-01

**Authors:** Tonantzin Samara Martinez-Lucio, Remco J. J. Knol, Oscar I. Mendoza-Ibañez, Lars van Wunnik, Friso M. van der Zant, Charalampos Tsoumpas, Riemer H. J. A. Slart, Sergiy V. Lazarenko

**Affiliations:** 1 Department of Nuclear Medicine and Molecular Imaging, University Medical Center Groningen, University of Groningen, 9713 GZ Groningen, The Netherlands; 2 Department of Nuclear Medicine, Northwest Clinics Alkmaar, 1815 JD Alkmaar, The Netherlands; 3 Department of Biomedical Photonic Imaging, University of Twente, 7522 NB Enschede, The Netherlands

**Keywords:** positron emission tomography, coronary artery disease, left ventricular function, left ventricular geometry, CardioFreeze, data-driven motion correction

## Abstract

**Background/Objectives**: Gated cardiac positron emission tomography (PET) synchronizes PET data to the cardiac cycle based on an electrocardiogram (ECG) signal, providing left ventricular (LV) functional and geometrical parameters. Nevertheless, image artifacts, due to cardiac-, breathing-, and/or patient-motion occurring during image acquisition, undermine the reliability and clinical utility of these parameters. This study aims to elucidate the effect of two motion correction (MC) tools, CardioFreeze (CF) and a data-driven motion correction (DDMC) prototype, on LV functional and geometrical parameters. **Methods**: ECG-gated rest/stress [^13^N]NH_3_ PET/CT scans from forty patients with myocardial ischemia and thirty-nine patients with normal myocardial perfusion were included. The following four reconstructions were performed for each patient scan: without motion correction (NMC), with CF, DDMC, and DDMC & CF. Images were processed with Cedars-Sinai QPET software. **Results**: End-diastolic volume (EDV) in rest and stress increased significantly using DDMC. End-systolic volume (ESV) increased significantly, while LV ejection fraction (LVEF) decreased significantly using any MC tool, regardless of the phase. Shape index end-systole (SI ES) and shape-index end-diastole (SI ED) increased significantly when using MC, except for SI ED in rest, where DDMC did not cause any difference. Eccentricity index end-systole (ECC ES) in rest and stress increased significantly in patients with normal myocardial perfusion, while it did not differ in ischemic patients after applying MC. **Conclusions**: MC tools significantly increase ESV values and decrease EF values. The highest effect is observed with the combined use of DDMC & CF. Image quality is greatly improved when using MC, regardless of the method, particularly in patients with the highest myocardial displacement.

## 1. Introduction

Myocardial perfusion imaging (MPI) PET is a powerful non-invasive method for diagnosis, therapy guidance, and prognosis of major adverse cardiac events in patients with coronary syndromes [1]. Advantages of this imaging modality compared with SPECT MPI include superior image quality and higher spatial and temporal resolution, resulting in greater diagnostic accuracy. Furthermore, PET MPI enables the reconstruction of dynamic images for absolute myocardial blood flow quantification [2].

In addition to the aforementioned importance, the electrocardiogram (ECG)-gated MPI PET series can evaluate the left ventricular (LV) function and geometry by estimating values of end-systolic volume (ESV), end-diastolic volume (EDV), ejection fraction (LVEF), shape index in end-systole (SI ES) and end-diastole (SI ED), and eccentricity index in end-systole (Ecc ES). ECG-gated PET is based on synchronizing the PET data with the heart’s electrical signal obtained with the ECG. PET data are subdivided into a certain number of equal-duration gates (i.e., eight or 16 gates) that correspond to different phases of the cardiac cycle [3,4]. Each gate-data are stored separately, and, when reconstructed, the gates of each phase are summed to the corresponding ones along the acquisition to obtain the final images from each phase of the cardiac cycle that will be ultimately used to estimate LV volumes and geometrical variables. LV assessment by ECG-gated MPI PET/CT has been demonstrated to be crucial in the diagnosis and prognosis of patients with coronary artery disease (CAD) [5,6,7,8,9].

Similar to the static- and dynamic-PET series, the ECG-gated series is impaired by motion from various sources (cardiac, contraction, respiration, and voluntary patient movement) during the scan. Although separating the acquisition data into independent gates corresponding to different phases of the cardiac cycle is already a method for mitigating motion due to cardiac contraction, this approach reduces the number of counts per frame, thereby impairing the quality of the final gated images. Furthermore, other sources of motion, primarily respiratory motion, can still degrade individual frames, causing mainly blurring of the myocardial wall [10].

Diverse motion correction (MC) methods have been developed for MPI PET/CT. These solutions can be primarily categorized into within-reconstruction and post-reconstruction methods, according to how they perform MC. Within-reconstruction methods are preferable, as they produce final images with less noise and bias than post-reconstruction approaches [11], while maintaining the inherent benefits of MC, such as better accuracy, improved image quality [12], and better agreement between software packages [13]. Post-reconstruction methods are generally time-consuming and lead to higher bias due to limited count-statistics [14]. They are also operator-dependent when implemented manually, and usually only allow MC to be performed on one outcome series [15].

Specifically in ECG-gated MPI PET, most of the solutions aimed to correct for respiratory motion have been based on external hardware systems, such as respiration sensors [16] and optical video cameras [12,17]. However, these methods are difficult to implement in clinical practice due to the complexity of setting up routine clinical protocols with the use of external motion-tracking devices. More recently, data-driven MC methods that work at the list-mode PET raw data level have been proposed and implemented [18]. Despite the implementation of motion correction methods in PET, the study of their effect on ventricular functional and geometrical parameters remains scarce.

With all the above and considering the availability of two MC methods in Northwest Clinics Alkmaar, the objective of this study was to evaluate the effect of the use of three different MC approaches on LV functional and geometrical variables. Additionally, myocardial motion vectors were analyzed to elucidate the prevalence and characteristics of myocardial motion during the period of image acquisition used for gated image reconstruction.

## 2. Materials and Methods

### 2.1. Study Population

The study population consisted of thirty-nine patients categorized as those with normal myocardial perfusion, also referred to as normal patients in the rest of the manuscript, and forty patients categorized as positive for myocardial ischemia, after undergoing an ECG-gated [^13^N]NH_3_ MPI PET/CT examination due to suspicion of ischemic heart disease (IHD) at the Northwest Clinics Alkmaar in Alkmaar, The Netherlands, from July 2020 to February 2022. Patients provided written informed consent for the use of their anonymized data. No additional measures or procedures were carried out on the patients, other than the standard PET/CT procedure and clinical management. The study was approved by the institutional research board; approval of the local ethical committee was not necessary since the study does not fall within the scope of the Dutch Medical Research Involving Human Subjects Act (Section 1.b, wet medisch-wetenschappelijk onderzoek [WMO], 26 February 1998).

### 2.2. Diagnostic Criteria

The following criteria were used to categorize patients as having normal myocardial perfusion: (1) no history of IHD; (2) PET/CT results interpreted as normal by an experienced nuclear medicine physician, i.e., no significant visual perfusion defects combined with summed rest score (SRS) and summed stress score (SSS) < 5, stress myocardial blood flow (MBF) > 1.85 mL/g/min, and coronary flow reserve (CFR) > 2.0, absence of coronary artery calcification, as determined by visual inspection of attenuation correction CT (ACCT) scans; (3) IHD diagnosis ruled out by medical consensus after an integral assessment (clinical history, symptomatology, and non-invasive tests); and (4) follow-up from scan date to inclusion date without the presence of major adverse cardiac events (MACEs).

The following criteria were used to classify patients as those with myocardial ischemia: (1) PET/CT results interpreted as abnormal by an experienced nuclear medicine physician, i.e., presence of perfusion defects by visual assessment and/or SSS > 5, stress MBF < 1.85 mL/g/min, and/or CFR < 2.0, presence of coronary artery calcification, as determined by visual inspection of ACCT scans; and (2) IHD diagnosis confirmed by medical consensus after an integral assessment consisting of clinical history, symptomatology, non-invasive tests, and a follow-up of at least 2 years.

The PET/CT reconstructions used for the patients’ diagnosis and classification in this project were not necessarily motion-corrected. MC was applied when artifacts due to motion were suspected, as recommended by the current guidelines [19].

### 2.3. Image Acquisition

Images were acquired using list-mode acquisition with a Biograph Vision 600 PET/CT scanner (Siemens Healthcare, Knoxville, TN, USA). Patients were instructed to refrain from consuming caffeine and xanthine products for 24 h before the scan. A time-efficient protocol was used, consisting of a 12 min rest acquisition and a 12 min stress acquisition. Rest acquisition started simultaneously with the intravenous administration of 300 MBq of [^13^N]NH_3_ (3 mL [^13^N]NH_3_, followed by 20 mL of NaCl at a rate of 0.4 mL/s, and flushed with 20 mL of NaCl at a rate of 2.0 mL/s). Immediately after the end of the rest acquisition, pharmacologic stress was induced either by an adenosine infusion (0.14 mg/kg/min for 6 min) or by a single bolus of 400 µg regadenoson through a second line. One minute after the end of rest acquisition, the stress imaging acquisition is started. After 2 min of the start of the stress acquisition, a second dose of 400 MBq of [^13^N]NH_3_ is infused (3 mL [^13^N]NH_3_, followed by 20 mL of NaCl at a rate of 0.4 mL/s, and flushed with 20 mL of NaCl at a rate of 2.0 mL/s).

### 2.4. Image Reconstruction and Processing

Static, 16-bin ECG-gated, and dynamic images were reconstructed from list-mode data using PSF + TOF reconstruction, a 220 × 220 matrix, zoom 2, an isotropic Gaussian 3D filter of 4 mm, 4 iterations, and 5 subsets. Decay, scatter, CT-based attenuation, and random corrections were applied to the images. Registration between PET and low-dose CT was evaluated and manually corrected for misalignment before reconstruction.

Dynamic rest images were reconstructed from the first 10 min of the rest acquisition data into 25 frames (1 × 10, 12 × 5, 2 × 10, 7 × 30, 2 × 60, and 1 × 180 s). Dynamic stress images were reconstructed using the last 10.5 min of data from stress, binned into 26 frames (1 × 30, 1 × 10, 12 × 5, 2 × 10, 7 × 30, 2 × 60, and 1 × 180 s) with the use of a residual activity correction (RAC) algorithm, as described by Opstal et al. [20]. This RAC algorithm quantifies the residual activity from the [^13^N]NH_3_ injection at rest using the first dynamic 30 s frame and subtracts this residual activity from every other frame in the dynamic images.

Static and gated rest images were acquired from the reconstruction of data from 2.5 min to 12 min into a single gate and 16 gates, respectively. Static and gated stress images were reconstructed using data from 4.5 min to 12 min, into 1 and 16 gates, respectively. Both static and gated stress images were reconstructed by applying the RAC algorithm, which quantified residual activity from 120 s before the second [^13^N]NH_3_ injection.

Gated images were reconstructed using the following different methods: (1) conventional ECG gating and (2) CardioFreeze (CF) (Siemens Healthineers, PETsyngo version VG80B), (3) data-driven motion correction (DDMC) (Siemens Healthineers, version 2023a), and (4) DDMC combined with CF (DDMC & CF). Image processing was performed with Quantitative Gated SPECT (QGS) software (Cedars-Sinai Cardiac Suite, Los Angeles, CA, USA [version: 2018.0.0.232]).

### 2.5. CardioFreeze

CardioFreeze is a within-reconstruction method that corrects for respiratory and/or cardiac motion via single or dual cardiac gating (cardiac and respiratory). This software is embedded in the PET reconstruction software (PETsyngo version VG80B) used for image reconstruction. This method performs MC with a two-step dual gating approach. In the first step, the list-mode raw data file is analyzed by the software to retrieve the respiratory signal for gating. In the second step, the cardiac signal from the ECG is used for cardiac gating purposes. In the end, the PET data are sorted into eight or sixteen cardiac gates and eight respiratory gates for each cardiac gate. Afterwards, a deblurring algorithm, based on a mass-conservation optical flow method, allows the reconstruction of each image, including the total number of counts in the whole acquisition, resulting in improved quality images. This deblurring step is processed using an optical flow method which involves the estimation of myocardial motion by following the elastic transformation between images in different gates and transforming images from all other gates to a preselected target gate [18,21,22].

### 2.6. Data-Driven Motion Correction

Armstrong et.al. and Hayden. et al. published a full explanation of the DDMC algorithm [13,23]. As a concise description, the DDMC algorithm uses the PET raw list-mode data to bin the position of the positron annihilation events into a 3D volume called the direct volume histogram (DVH). A DVH frame is created for every second of the acquisition. One DVH is selected as the reference DVH. This reference is a DVH from the tissue phase (scan period with the highest myocardial uptake and the lowest extracardiac activity) which takes place after 180 s. After the DVH creation, the DDMC prototype tracks the myocardial wall motion during the total acquisition time by comparing every DVH to the reference. A single motion vector is produced in each spatial axis as follows: *X* reflecting anterior–posterior motion, *Y* reflecting latero-lateral motion, and *Z* reflecting craniocaudal motion. Finally, the motion vector in the *Z*-axis is subsampled and used during the sinogram rebinning process to produce a motion-corrected sinogram, which is then reconstructed using regular protocols.

### 2.7. DDMC & CF

The effect of the combined use of both MC algorithms was also evaluated, as each of the algorithms is applied at different steps during image reconstruction and corrects different types of motion (DDMC correcting rigid cardiac motion caused by breathing and gross-patient motion, and CF correcting mainly elasting myocardial motion occurring between breathing and cardiac phases). Therefore, the methods could be used complementary to one another. To this end, first, the original list-mode raw data file was processed with the use of DDMC prototype. In the second step, when reconstructing the PET-gated series, the dual ECG-gated with CardioFreeze was enabled.

### 2.8. Left Ventricular Function

LV volumes and EF were obtained from ECG-gated images at rest and stress with QGS software. This software uses a 3D volume-based method, in which the LV volumes are delimited by the valve plane and the endocardium for each interval in the cardiac cycle. End-systolic volume (ESV) and end-diastolic volume (EDV), stroke volume (SV), and LVEF are automatically calculated [24,25].

### 2.9. Left Ventricular Geometry

Shape index (SI) is defined as the ratio between the maximum 3D short- and long-axis dimensions from the left ventricle at end-diastolic and end-systolic frames. The ratio is expressed from 0 to 1, with 0 being an ellipsoid and 1 a sphere, corresponding to the LV shape [26]. On the other hand, eccentricity index (EI) is a measure of LV elongation and ranges from 0 to 1, with 0 being a sphere and 1 an ellipsoid form of the left ventricle [27,28]. Both indices are calculated with QGS software.

### 2.10. Myocardial Rigid Motion

The myocardial motion vectors drawn by the DDMC were obtained to analyze the prevalence and characteristics of myocardial motion during the acquisition period used for reconstruction of gated images. The myocardial positions per second were converted to absolute terms and assigned to one of the following categories of myocardial shift: 0 to 3 mm, 3 to 6 mm, 6 to 9 mm, 9 to 12 mm, and higher than 12 mm to elucidate the prevalence of motion of different intensities in individual scans. A 3 mm classification was chosen, as the PET scanner used for this project has an axial and transverse spatial resolution of 3.5 and 3.6 mm, respectively [29]. Furthermore, these positions were averaged to obtain a single metric of myocardial displacement (MyoDis) in each axis (*X*, *Y*, and *Z*) for every scan. Differences in MyoDis between axes and scan phases were tested using Kruskal–Wallis and Mann–Whitney U tests, respectively. Finally, a threshold of 30% of the total reconstructed time with myocardial shift > 6 mm in the *Z*-axis was used to classify stress scans as with “High Motion” (>30%) or “Low Motion” (<30%), to explore whether the effect of MC increased with the scan motion burden, and to isolate examinations with the highest motion for visual inspection before and after MC. The shift in the *Z*-axis was selected as this is the direction used by the DDMC prototype for performing MC. The threshold of 30% was selected arbitrarily, as it was a cut-off value that allowed for a balanced number of scans in both categories of “Low Motion” and “High Motion”, with an approximate ratio of 60/40, respectively, in both groups. Rest scans were excluded due to the overall low prevalence of motion larger than 6 mm.

### 2.11. Statistical Analysis

Normality tests were conducted using the Shapiro–Wilk test and Q-Q plot. Normally distributed categorical values were expressed using frequencies and percentages, whereas continuous values were presented with their mean ± standard deviation (SD) values. Baseline characteristics, risk factors, and cardiovascular history variables were compared as proportions with χ^2^. Differences in left ventricular functional and geometrical variables between MC methods, as well as the perfusion status, were analyzed with a two-way repeated measures ANOVA, using MC methods (NMC, CF, DDMC, and DDMC & CF) as within-subject factors, and perfusion group (normal perfusion and ischemia) as between-subjects factor. The model was applied independently to variables at rest and at stress. To control the higher error rate when running multiple comparisons, significance levels were corrected using the Bonferroni correction. Furthermore, an additional correction (i.e., Greenhouse–Geisser correction) was used in the model for the variables that violated the assumption of sphericity. When a significant *p*-value (<0.05) was observed with the two-way repeated measure ANOVA, a pairwise comparison based on the estimated marginal means was used to find the specific group(s) that differed. Statistical analyses were executed using IBM SPSS Statistics version 31.0 (Armonk, NY: IBM Corp., USA), where two-tailed *p*-values were considered statistically significant. Graphs were performed with GraphPad Prism 9.1.

## 3. Results

### 3.1. Patients’ Characteristics

Table 1 shows the characteristics of the study population. The overall cohort consisted mainly of women (51.9%), with a mean age of 79 years. Several statistical differences, in line with the underlying diagnosis, were found between the group of normal patients and patients with myocardial ischemia. Women constituted 74.4% of the normal perfusion patients, whereas this proportion was much lower (30%) in the ischemic group. Also, the prevalence of hypercholesterolemia, previous myocardial infarction, prior percutaneous coronary intervention, and values of the coronary artery calcium score were higher in the group of patients with ischemia. Due to differences in gender prevalence across groups, an independent *t*-test was used to compare variables of interest between men and women. As expected, all LV functional parameters differed significantly between genders. Males showed higher volumes and lower LVEF, independently of the MC tool applied. Regarding LV geometry, SI ES was significantly different between genders with NMC, independently of the phase. Similarly, SI ES stress was significantly higher in males when applying CF, DDMC, and DDMC & CF. These results are presented in Appendix A, Table A1 and Table A2.

### 3.2. Effect of Motion Correction Tools on Left Ventricular Function Quantification

Between-subject analysis showed that EDV, ESV, and LVEF values were significantly different in ischemic patients than in normal patients, regardless of the phase scan or the use of MC (*p* < 0.05). These results can be found in Appendix A, Table A3. Similarly, within-subject analysis revealed that the use of MC had a significant effect on values of EDV, ESV, and LVEF, irrespective of the phase and patient group (*p* < 0.001).

**Figure 1:**
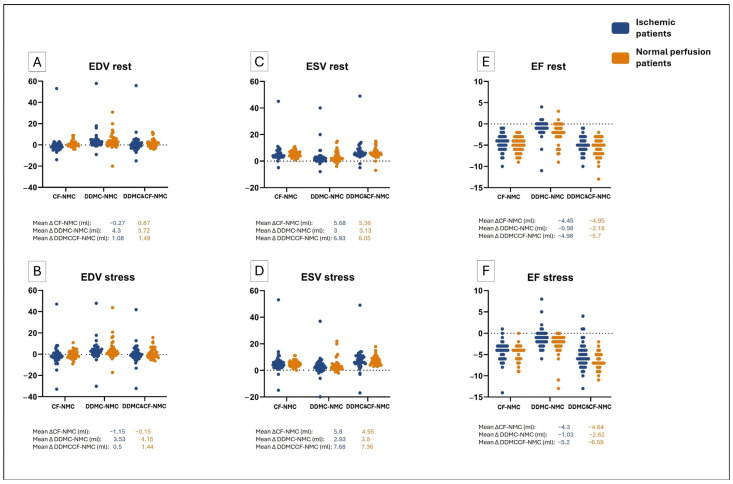
Difference plots of LV functional variables. Every single point illustrates the difference in value after the use of MC for the LV functional variables ((**A**). EDV in rest, (**B**). EDV in stress, (**C**). ESV in rest, (**D**). ESV in stress, (**E**). LVEF in rest, (**F**). LVEF in stress). Note the consistently positive differences in ESV showing a significant increase with each MC tool. Meanwhile, the differences from LVEF tend to be below the 0 line.

Table 2 shows the mean values of LV functional parameters in rest and stress for both patient groups. Accordingly, Figure 1 illustrates the differences in values of EDV, ESV, and LVEF after the use of each MC method when compared to NMC values. These differences are shown for every patient group and for variables acquired in rest and in stress.

End-Diastolic Volume. Post hoc pairwise analysis demonstrated that values of EDV rest and EDV stress statistically differed only when using DDMC. As shown in Table 2, the highest mean EDV values were found when using DDMC. Also, as observed in Figure 1A,B, DDMC led to a difference in EDV of at least +3.53 mL in stress and at least +3.73 mL in rest, regardless of the patient group. With respect to other MC approaches, the use of DDMC & CF caused non-significant increases in EDV values, regardless of the phase and patient group, with a maximum increase of +1.49 mL. Finally, CF had non-significant heterogeneous effects on EDV values.

End-Systolic Volume. Pairwise comparison analysis indicated that ESV differed significantly after the use of any motion correction method (*p* < 0.05). More specifically, values of ESV always increased when using MC tools, regardless of the phase and patient group. Additionally, the effect on ESV was significantly different between all MC tools. As shown in Figure 1C,D and Table 2, the use of DDMC & CF had the highest effect, from +6.05 mL to +7.68 mL, and presented the highest mean EDV values. Similarly, the use of CF led to an EDV increase between +4.95 mL and 5.8 mL. Finally, DDMC caused the smallest increments, between +2.93 mL and +3.8 mL. All effects of the use of MC tools were similar in normal and ischemic groups.

Left ventricular ejection fraction. Post hoc analysis revealed that LVEF changed significantly when using MC tools (*p* < 0.005). However, the effect of MC was different for LVEF in rest and in stress. In the case of LVEF in rest, the use of any MC tool decreased LVEF values in rest. Furthermore, the effect of each MC tool differed significantly from each other. More specifically, DDMC & CF produced the greatest decrement in LVEF in rest, with changes ranging from −4.98% to −5.7%, followed by CF, with decrements ranging from −4.45% to −4.95%, and DDMC, with decrements between −0.98% and −2.18% (Figure 1E). In the case of LVEF in stress, the analysis demonstrated that the effect of MC differed between the groups of normal and abnormal patients. In both groups, the use of MC led to a decrease in LVEF in stress values. However, in patients with normal perfusion, all these decrements were statistically significant, and additionally, the effects of every MC approach differed significantly from each other. On the contrary, in patients with ischemia, the decrease caused by using DDMC was not significant, and the effect of CF and DDMC & CF did not differ. This difference is illustrated in Figure 1F, where it can be observed that in the group of normal patients, the DDMC mean difference was −2.62% compared to −1.03% in the ischemic group. Likewise, in the ischemic group, the gap between the DDMC & CF and CF mean differences was 0.9%, compared to the gap of 1.95% in the normal group.

### 3.3. Effect of Motion Correction Tools on Left Ventricular Geometry

Between-subject analysis demonstrated that SI ED, SI ES, and Ecc ES values were significantly different in ischemic than in normal patients, regardless of the phase scan or the use of MC (*p* < 0.05), except for SI ED rest and Ecc ES rest. These results can be found in Appendix A, Table A4. In the same way, within-subject analysis revealed a significant effect of MC on values of SI ED, SI ES, and Ecc ES, irrespective of the phase and patient group (*p* < 0.001).

**Figure 2:**
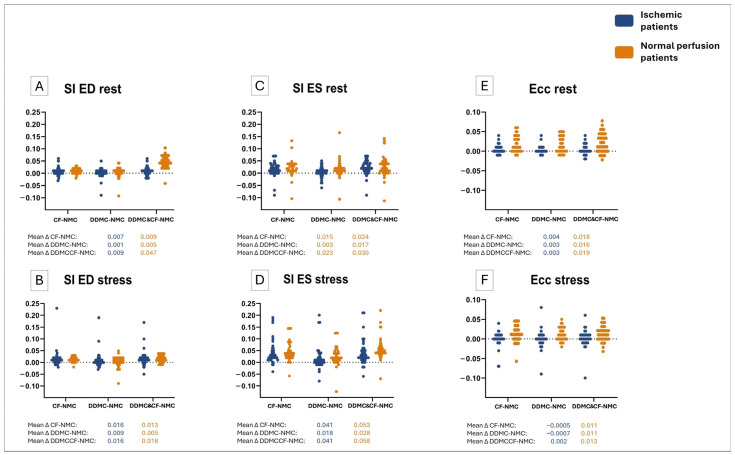
Difference plots of LV geometrical variables. Every single point illustrates the difference in value after the use of MC for the LV geometrical variables ((**A**). SI ED in rest, (**B**). SI ED in stress, (**C**). SI ES in rest, (**D**). SI ES in stress, (**E**). Ecc ES in rest, (**F**). Ecc ES in stress). Note how the differences in SI ES and SI ED are above the 0 line, reflecting a significant increase when applying MC. The differences in Ecc ES from ischemic patients are closer to the 0 line, while Ecc ES are above the 0 line.

Table 3 shows the mean values of LV geometrical parameters in rest and stress for both patient groups. Similarly, Figure 2 contains the difference plot for every LV geometrical variable, illustrating the differences in each value after the use of each MC method when compared to NMC values. These differences are shown for every patient group and for variables acquired in rest and in stress.

Shape index end-diastolic. Post hoc analysis revealed that the use of DDMC did not affect SI ED values in rest, whereas CF and DDMC & CF significantly increased SI ED in rest. On the other hand, all MC tools statistically increased SI ED during stress. Nevertheless, the overall effect of MC in SI ED was small, ranging from 0.1% to 4.7%. The effect of MC in SI ED was similar in normally perfused patients and in patients with myocardial ischemia. It can be seen in Figure 2 (panels A and B) that the highest difference on SI ED either in rest or stress was with DDMC & CF.

Shape index end-systolic. The use of any MC tool proved to have a significant effect on SI ES value. All methods significantly increased SI ES, regardless of the patient group and scan phase. Within this parameter, DDMC & CF led to the highest mean SI ES values, as depicted in Table 3, and the highest effect, as shown in Figure 2 (panels C and D), ranging from +0.023 to +0.058 (2.3–5.8%). CF followed DDMC & CF, and lastly, DDMC demonstrated the lowest increments in SI ES. Similarly to what was observed in SI ED, the overall MC effect was a minor increase in SI ES, independent of the phase and group of patients. However, there was not significant difference between DDMC and NMC SI ES values, either at rest or under stress.

Eccentricity index. The post hoc analysis in Ecc ES revealed a significant effect of using MC tools. Additionally, both for Ecc ES in rest and in stress, the effect of using MC differed significantly between patients with and without myocardial ischemia. More specifically, in the group of normal patients, the use of any MC method significantly increased Ecc ES during both phases. On the other hand, in patients with ischemia, the use of MC did not significantly modify Ecc ES values, either in rest or stress. As observed in Figure 2E,F, the larger differences in Ecc ES were obtained with the use of DDMC & CF (+0.13 to +0.19), while changes derived from the use of DDMC and CF were fairly similar.

### 3.4. Prevalence and Characteristics of Myocardial Rigid Motion

**Figure 3:**
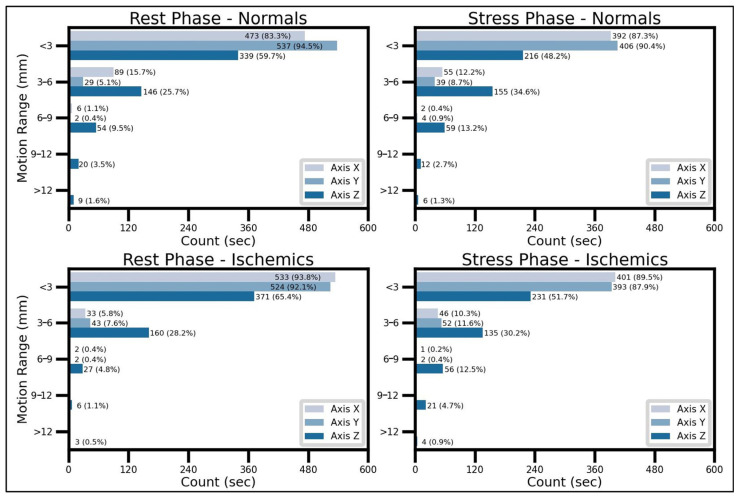
Mean amount of motion of different intensity ranges in the 3D space. Motion predominantly occurs along the Z axis within the range of 3–6 mm and above, with a higher prevalence during the stress phase.

After analyzing the motion vectors provided by the DDMC prototype, it was found that motion was substantial during the acquisition time used for reconstructing ECG-gated PET/CT scans, particularly in the craniocaudal direction (*Z*-axis). As observed in Figure 3, regardless of the scanning phase or patient group, the myocardial wall shifts, on average, less than 3 mm during at least 83% of the total reconstruction time in the anterior–posterior (*X-axis*) and latero-lateral (*Y-axis*) directions. However, this percentage drops drastically to less than 66% in rest examinations and 52% in stress scans in the *Z* axis. Interestingly, motion higher than 6 mm is practically negligible in the *X* and *Y* axes, accounting for an average of less than 1.2% of reconstruction time. Conversely, craniocaudal motion exceeding 6 mm ranged from 6.4% (rest phase from ischemic patients) to 18.1% (stress phase from ischemic patients) of the reconstructed time.

**Figure 4:**
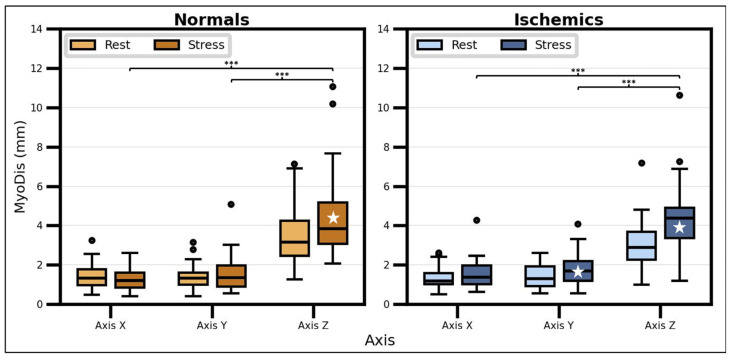
Myocardial displacement (MyoDis) per phase in the 3D space. MyoDis is significantly higher in the Z axis regardless of the patient group and phase. The white stars indicate where MyoDis was significantly higher at stress versus at rest condition. ***: *p* < 0.001.

Figure 4 illustrates the differences in MyoDis between axes and phases. After statistical analysis, it was found that the MyoDis in the Z axis was significantly higher than in the *X* and *Y* axes, regardless of the phase and patient group. When differences between phases were tested, it was found that MyoDis was significantly higher in stress than in rest in the *Z* axis of both normal and ischemic patients. MyoDis also proved to be statistically higher during stress in the Y axis of the ischemic group; nevertheless, in this axis, the MyoDis remained small with median values < 2 mm, irrespective of the group.

**Figure 5:**
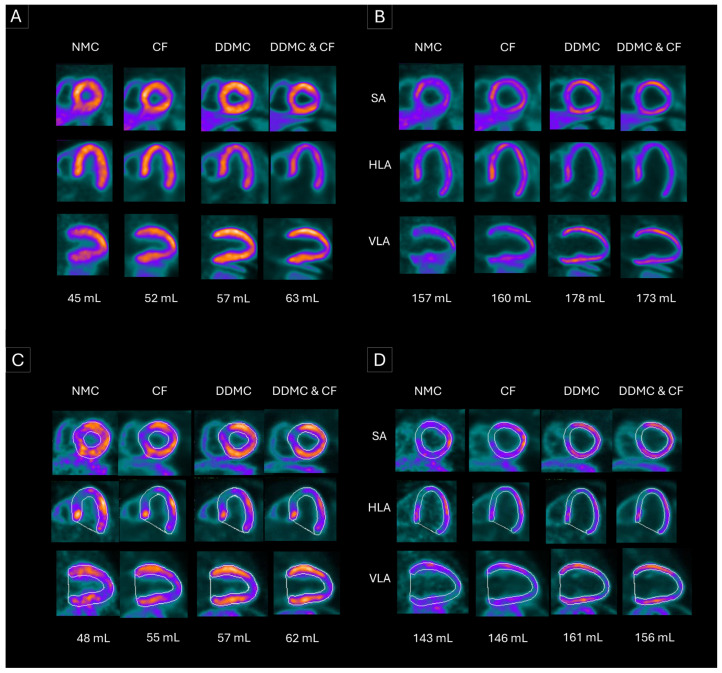
Screenshots of SA, HLA, and VLA at end-systole (**A**,**C**) and end-diastole (**B**,**D**) with NMC, CF, DDMC prototype, and DDMC & CF. These images correspond to the scans in stress from two patients from our cohort. (**A**,**B**) correspond to a patient with normal myocardial perfusion, and (**C**,**D**) correspond to a patient with myocardial ischemia. When MC is applied, the myocardial wall becomes thinner, images are sharper, and there is a better contrast of the myocardial wall to the LV. SA: short axis, HLA: horizontal long axis, VLA: vertical long axis, NMC: no motion correction, CF: CardioFreeze, DDMC: data-driven motion correction.

**Figure A1:**
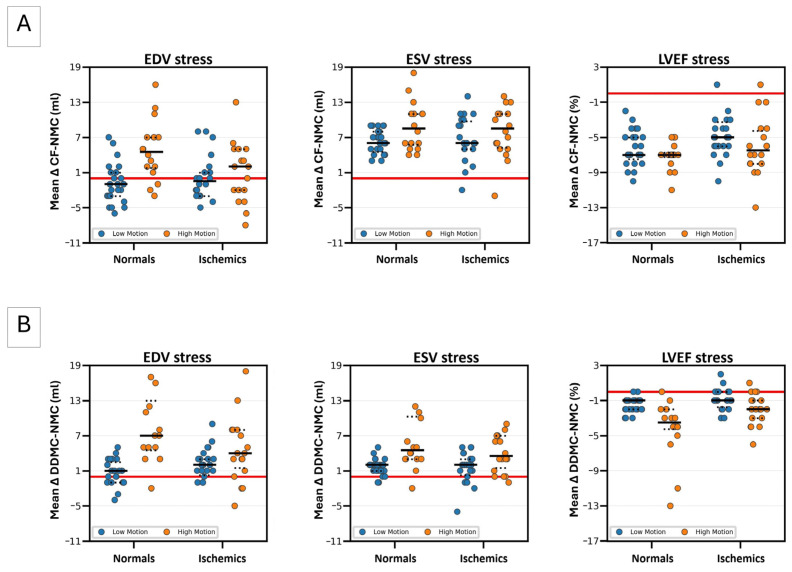
Mean differences in each LV geometrical variable in stress between NMC and CF (**A**), and DDMC (**B**), divided into groups according to the amount of motion (low and high) and the perfusion status (normal perfusion and ischemic). Note that the mean differences in patients with high motion are too high compared to the mean values of patients with low motion. This reflects that both correction methods have a greater effect on LV function variables when patients present more motion. LV: left ventricular, NMC: no motion correction, CF: CardioFreeze, DDMC: data-driven motion correction.

Our exploratory analysis suggests that both DDMC and CF correct EDV, ESV, and LVEF values to an extent related to the amount of motion present in scans. This is illustrated in Appendix A, Figure A1, which shows that larger differences in LV volumes and LVEF are observed when using MC in scans with high motion, regardless of the MC method or the patient group. Finally, the visual inspection of the final gated images of patients with the highest scan motion revealed a knock-on effect of motion, as exemplified in Figure 5. First, when motion is substantial, the myocardial wall suffers from image blurring, consequently increasing the thickness of the myocardial wall, particularly in the end-systolic phase. This results in an inaccurate delineation of the endocardial contours, ultimately leading to artificially lower LV values and higher LVEF values.

## 4. Discussion

In this study, we examined the effect of the use of MC in the estimation of LV functional and geometrical parameters. Furthermore, we have objectively measured the amount of myocardial rigid motion present during the acquisition time of ECG-gated PET/CT examinations and explored the relation of scan motion and change in LV functional values after MC. Our main findings show that the use of MC tools in ECG-gated [^13^N]NH_3_ MPI PET/CT examinations improves image quality by removing image artifacts and myocardial blurring, enables sharp delineation of the endocardial contours, and ultimately significantly modifies the LV functional and geometrical values. Regarding LV function estimation, we have observed that EDV and ESV values tend to increase; therefore, LVEF values tend to decrease after using MC. We hypothesize that the increase in EDV and ESV after MC is due to the sharper delineation of the myocardial contours, which allows a more accurate calculation of LV volumes. With respect to LV geometrical variables, all values have increased after applying MC. Comparing our findings with the previous literature is difficult, as publications with similar objectives are scarce. Moreover, the small number of publications have evaluated MC in ECG-gated PET mainly with simulated data and a few clinical datasets obtained from healthy volunteers or with PET/CT acquisition protocols other than routine clinical MPI PET/CT examinations. Finally, to our knowledge, our study is first to evaluate the effect of MC on the estimation of LV geometrical variables.

Despite the above, we have found that our findings regarding LV volumes align with the findings reported by Tang et al. In their study, they have investigated the effect of respiratory MC on LV functional parameters. Similar to us, they have reported that LV volumes increased after respiratory MC [30]. However, with respect to LVEF, MC impacted differently depending on whether myocardial wall contraction was preserved. They described that LVEF values increased in patients with normal myocardial contraction, while remaining unchanged in cases with impaired myocardial contractility. Our cohort included both patients with normal perfusion and with myocardial ischemia, but we have not observed a different effect of MC, as in both groups, LVEF decreased after applying MC. Nevertheless, we did not collect information on wall motion scores, and the classification of patients was not only based on LVEF values, but also on an overall assessment of patient history, semi-quantitative scores, and myocardial blood flow values. Finally, Tang et al.’s results were obtained by using mainly simulated data and only two [^18^F]FDG cardiac PET scans, a factor that limits the extrapolation of their results.

The second publication that has evaluated a data-driven respiratory motion compensation technique for MC of list-mode PET data is the one from Lassen et al. [31]. They have explored the feasibility of their method using simulated data and seven [^18^F]FDG/[^13^N]NH_3_ myocardial viability PET/MR scans. Like us, they have reported that the use of MC improves image quality and delineation of the left ventricular wall. However, they have reported a reduction in ESV and EDV values. These findings are contrary to what we have observed in our cohort; yet again, the factors of a small sample size and the use of myocardial viability scans limit the generalizability of their outcomes. An added value of the study by Lassen et al. is that they could perform a head-to-head comparison of LVEF values obtained by PET against those obtained from the gold-standard cardiac magnetic resonance (CMR). They have described that LVEF values correlate better after the use of MC, although not significantly. Moreover, the specific results were not presented in the publication or the supplementary material. Maurer et al. have also examined the LV functional values reproducibility between ECG-gated [^13^N]NH_3_ PET and CMR, although without applying any type of MC to their PET scans [32]. They have described a good agreement between the two image modalities, but also found a significant underestimation of LV volume values when measured by ECG-gated [^13^N]NH_3_ PET. With these recent reports from Maurer et al., we can hypothesize that using MC tools might be useful to mitigate these systematic differences, considering that we have demonstrated that ESV and EDV increased substantially after MC. Nevertheless, from our study, it cannot be concluded safely whether the use of MC improves the accuracy of LV measurements, as a reference method was not included. Further prospective studies that aim to evaluate the reproducibility of LV functional and geometrical variables by ECG-gated PET against CMR before and after using MC are needed.

We have observed differences in performance in relation to the specific MC method. For instance, CF and DDMC & CF did not significantly modify EDV values in either rest or stress, whereas DDMC was the only method that did not increase SI ED in rest. However, these differences are most probably related to the different functioning of CF and DDMC. Both methods are applied within the PET/CT reconstruction process as they work with list-mode raw data; however, CF uses the list-mode raw data for a data-driven estimation of the respiratory and cardiac gating, while performing MC at the final reconstructed gated images level. On the contrary, DDMC uses the list-mode file to estimate myocardial motion and uses the motion vector in the *Z* axis to construct a “motion-corrected” sinogram. Additionally, as the temporal resolution of the DVH is one second, DDMC does not correct for cardiac contraction, which happens on a sub-second resolution. Despite all these observations, both methods consistently pointed in the same direction in all cases where these proved to significantly modify any value, i.e., both methods increased or decreased the value of the variable in question. When both methods were applied, i.e., DDMC & CF, the MC effect seems to be higher, especially on ESV EF, by giving the highest and the lowest values, respectively. This can be explained by the fact that, by applying two different motion correction methods, the images become sharper and provide a more accurate depiction of the walls of the left ventricle. This consequently yields a volume measurement that is closer to the physiological value.

Regarding LV geometrical variables, the use of MC was demonstrated to affect these variables. However, the role of these variables by PET has not been fully evaluated, and there are no clinically validated thresholds for using these variables as diagnostic or prognostic markers. Additionally, given that these variables range from zero to one, the absolute value differences after using MC are substantially small, despite reaching statistical significance. With the above, it is not possible to elaborate on the true meaning of these findings, and further research is needed on these geometric parameters in PET. However, based on our results, future research must consider the influence of motion during the scan acquisition and the impact of MC on LV geometrical variables.

Finally, we have been able to objectively measure the amount of myocardial rigid motion present in routine clinical ECG-gated [^13^N]NH_3_ MPI PET/CT examinations by analyzing the motion vectors from the DDMC prototype. Our findings in this respect are in line with what has been previously reported for static and dynamic ^82^Rb/[^13^N]NH_3_ MPI PET/CT acquisitions, as we have observed substantial myocardial motion in the craniocaudal direction that is more prominent during stress acquisitions [23,33]. When compared to a previous finding made by Mendoza-Ibañez et al., regarding the amount of motion in dynamic acquisitions, also measured with the DDMC prototype, we have observed a lower percentage of acquisition time with myocardial motion larger than 6 mm [33]. This last observation could be explained by the fact that for reconstruction of ECG-gated images, the early phase (blood-pool phase) of the scan is excluded, and consequently, final images are not influenced by the stressor agents, which have been demonstrated to increase cardiac and respiratory motion, particularly if using the pharmacological agent adenosine.

## 5. Limitations

This study presents limitations that need to be addressed. Although the DDMC prototype tracks motion on all three axes, it only performs motion correction on the *Z-axis* to maintain computational efficiency. It can be the case with high motion along the antero-posterior and latero-lateral directions, in which the correction on the *Z-axis* may not be sufficient. However, in our cohort of 79 patients, we did not find any scan in which either the motion in *X* or *Y* axis would be relevant, or the DDMC was unsuccessful for improving image quality and myocardial contour detection. As mentioned in the Discussion, the absence of a reference standard method in our study prevents us from concluding the effect of MC on the agreement of PET-derived LV variables.

## 6. Conclusions

Motion correction considerably improves image quality and the detection of myocardial contours. More importantly, it modifies the values of LV function and geometry significantly. MC overcomes the underestimation of LV volumes, as described for ECG-gated [^13^N]NH_3_ PET/CT acquisitions, where substantial motion was demonstrated, particularly in the craniocaudal direction (*Z axis*) and during stress.

## Figures and Tables

**Table 1:** Baseline population characteristics.

Parameter	All N = 79	Normal Perfusion Patients n = 39	Ischemic Patients n = 40	*p* Value
**Age—mean years (SD)**	79 (10)	66 (11)	69 (8)	ns
**Women—n (%)**	41 (51.9)	29 (74.4)	12 (30)	<0.001
**Weight—mean kg (SD)**	80.1 (14.7)	78.5 (14)	81.6 (15.3)	ns
**Height—mean cm (SD)**	171.4 (10.4)	168.1 (11.3)	174.7 (8.4)	0.004
**BMI—mean (SD)**	27.4 (5.3)	28 (6)	26.7 (4.5)	ns
**Risk factors**				
**Smoking—n (%)**	6 (7.6)	3 (7.7)	3 (7.5)	ns
**Hypertension—n (%)**	40 (50.6)	17 (43.6)	23 (57.5)	ns
**Diabetes mellitus—n (%)**	7 (8.9)	2 (5.1)	5 (12.5)	ns
**Hypercholesterolemia—n (%)**	34 (43)	10 (25.6)	24 (60)	0.003
**Cardiovascular history**				
**Prior myocardial infarction—n (%)**	8 (10.1)	0 (0)	8 (20)	0.005
**Prior PCI—n (%)**	11 (13.9)	0 (0)	11 (27.5)	<0.001
**Prior CABG—n (%)**	2 (2.5)	0 (0)	2 (5)	ns
**Calcium in coronaries—n (%)**	58 (73.4)	21 (53.8)	37 (92.5)	<0.001

**Table 2:** LV functional variables with and without the application of MC.

Parameter	Normal Perfusion Patients n = 39				Ischemic Patients n = 40			
	NMC	CF	DDMC	DDMC & CF	NMC	CF	DDMC	DDMC & CF
**EDV in rest—mL** **[95% CI]**	103.5 [92.7, 114.2]	104.4 [93.3, 115.4]	106.6 [95.3, 117.9]	105 [93.9, 116]	126.4 [115.7, 137]	126.1 [115.2, 137]	130.7 [119.5, 141.8]	127.4 [116.5, 138.4]
**EDV in stress—mL** **[95% CI]**	107.3 [95.8, 118.7]	107.1 [95.7, 118.6]	110.7 [98.7, 122.6]	108.7 [97.3, 120.2]	137.3 [126, 148.6]	136.2 [124.9, 147.5]	140.9 [129, 152.7]	137.8 [126.5, 149.1]
**ESV in rest—mL** **[95% CI]**	29.5 [22.9, 36]	34.8 [27.6, 42.1]	32.2 [25.1, 39.2]	35.5 [28.2, 42.9]	47.8 [41.3, 54.2]	53.5 [46.3, 60.6]	50.8 [43.8, 57.7]	54.7 [47.7, 62]
**ESV in stress—mL** **[95% CI]**	26.7 [19.8, 33.6]	31.6 [23.9, 39.4]	29.7 [22.3, 37.1]	34.1 [26.3, 41.8]	53.4 [46.5, 60.2]	59.2 [51.5, 66.9]	56.3 [49, 63.6]	61 [53.4, 68.6]
**LVEF in rest—%** **[95% CI]**	72.6 [69.9, 75.2]	67.6 [64.9, 70.3]	70.7 [68.1, 73.3]	66.9 [64.3, 69.5]	64 [61.4, 66.6]	59.5 [56.9, 62.2]	63 [60.4, 65.6]	59 [56.5, 61.5]
**LVEF in stress—%** **[95% CI]**	76.3 [73.6, 79]	71.7 [68.9, 74.5]	74.2 [71.6, 76.8]	69.7 [67.1, 72.4]	62.7 [60.1, 65.4]	58.4 [55.6, 61.2]	61.7 [59.1, 64.3]	57.5 [54.9, 60.2]

**Table 3:** LV geometrical variables with and without the application of MC.

Parameter	Normal Perfusion Patients n = 39				Ischemic Patients n = 40			
	NMC	CF	DDMC	DDMC & CF	NMC	CF	DDMC	DDMC & CF
**SI ED in rest—unitless**	0.65 [0.64, 0.67]	0.66 [0.64, 0.68]	0.66 [0.64, 0.68]	0.66 [0.64, 0.68]	0.67 [0.65, 0.69]	0.68 [0.66, 0.69]	0.67 [0.65, 0.69]	0.68 [0.66, 0.7]
**SI ED in stress—unitless [95% CI]**	0.68 [0.66, 0.7]	0.69 [0.67, 0.71]	0.69 [0.67, 0.71]	0.70 [0.68, 0.72]	0.72 [0.7, 0.73]	0.73 [0.71, 0.75]	0.73 [0.71, 0.74]	0.73 [0.71, 0.75]
**SI ES in rest—unitless [95% CI]**	0.43 [0.41, 0.46]	0.46 [0.43, 0.48]	0.45 [0.43, 0.48]	0.46 [0.44, 0.49]	0.48 [0.46, 0.51]	0.50 [0.47, 0.52]	0.49 [0.46, 0.51]	0.51 [0.48, 0.53]
**SI ES in stress—unitless [95% CI]**	0.43 [0.41, 0.46]	0.49 [0.46, 0.52]	0.47 [0.44, 0.5]	0.49 [0.46, 0.52]	0.53 [0.5, 0.56]	0.57 [0.54, 0.6]	0.55 [0.52, 0.58]	0.57 [0.54, 0.6]
**Ecc ES in rest—unitless [95%CI]**	0.87 [0.86, 0.88]	0.88 [0.88, 0.89]	0.88 [0.87, 0.89]	0.88 [0.88, 0.89]	0.87 [0.86, 0.88]	0.87 [0.86, 0.88]	0.87 [0.86, 0.88]	0.87 [0.86, 0.88]
**Ecc ES in stress—unitless [95% CI]**	0.85 [0.84, 0.87]	0.87 [0.85, 0.88]	0.87 [0.86, 0.88]	0.87 [0.86, 0.88]	0.84 [0.83, 0.85]	0.84 [0.82, 0.85]	0.84 [0.82, 0.85]	0.84 [0.83, 0.85]

## Data Availability

The data presented in this study are available on request from the corresponding author due to privacy.

## Abbreviations

The following abbreviations are used in this manuscript:

CAD	Coronary artery disease
DDMC	Data-driven motion correction
DVH	Direct volume histogram
Ecc ES	Eccentricity index in end-systole
ECG	Electrocardiogram
EDV	End-diastolic volume
ESV	End-systolic volume
LV	Left ventricle
LVEF	Left ventricular ejection fraction
MC	Motion correction
PET/CT	Positron emission tomography/computed tomography
SI ED	Shape index in end-diastole
SI ES	Shape index in end-systole

## Appendix A

**Table A1:** Mean LV functional variables by MC group compared between genders.

	Parameter	Women n = 41	Men n = 38	*p* Value
**NMC**	End-diastolic rest (mL)	99.9 (24.4)	131.4 (38.4)	<0.001
	End-diastolic stress (mL)	104.9 (26.4)	141.5 (41.2)	<0.001
	End-systolic rest (mL)	29.2 (13.2)	49.1 (25.6)	<0.001
	End-systolic stress (mL)	29.0 (16.3)	52.3 (27.9)	<0.001
	Left ventricular ejection fraction rest (%)	71.8 (7.2)	64.3 (9.7)	<0.001
	Left ventricular ejection fraction stress (%)	73.9 (9.6)	64.7 (10.2)	<0.001
**CF**	End-diastolic rest (mL)	99.9 (23.8)	132 (39.7)	<0.001
	End-diastolic stress (mL)	104.6 (25.5)	140.4 (41.9)	<0.001
	End-systolic rest (mL)	34.0 (13.8)	55.3 (28.7)	<0.001
	End-systolic stress (mL)	33.8 (16.8)	58.3 (31.9)	<0.001
	Left ventricular ejection fraction rest (%)	66.9 (7.1)	59.8 (99.9)	<0.001
	Left ventricular ejection fraction stress (%)	69.0 (9.7)	60.7 (10.8)	<0.001
**DDMC**	End-diastolic rest (mL)	102.6 (24.1)	136.3 (41.1)	<0.001
	End-diastolic stress (mL)	107.9 (27.5)	145.4 (43.0)	<0.001
	End-systolic rest (mL)	31.5 (13.3)	52.4 (27.9)	<0.001
	End-systolic stress (mL)	31.7 (16.8)	55.5 (29.6)	<0.001
	Left ventricular ejection fraction rest (%)	70.1 (6.7)	63.2 (9.8)	<0.001
	Left ventricular ejection fraction stress (%)	71.9 (9.2)	63.5 (9.8)	<0.001
**DDMCCF**	End-diastolic rest (mL)	100.8 (23.6)	133.2 (40.1)	<0.001
	End-diastolic stress (mL)	105.8 (26.1)	142.5 (41.1)	<0.001
	End-systolic rest (mL)	34.8 (13.7)	56.5 (29.2)	<0.001
	End-systolic stress (mL)	36.0 (17.2)	60.3 (31.1)	<0.001
	Left ventricular ejection fraction rest (%)	66.2 (6.8)	59.3 (9.7)	<0.001
	Left ventricular ejection fraction stress (%)	67.3 (9.3)	59.5 (10.1)	<0.001

**Table A2:** Mean LV geometrical variables by MC group compared between genders.

	Parameter	Women n = 41	Men n = 38	*p* Value
**NMC**	Shape index end-diastolic rest (-)	0.66 (0.06)	0.67 (0.05)	ns
	Shape index end-diastolic stress (-)	0.69 (0.07)	0.70 (0.05)	ns
	Shape index end-systolic rest (-)	0.44 (0.08)	0.48 (0.07)	0.03
	Shape index end-systolic stress (-)	0.46 (0.10)	0.51 (0.08)	0.02
	Eccentricity index rest (-)	0.86 (0.03)	0.87 (0.03)	ns
	Eccentricity index stress (-)	0.84 (0.04)	0.85 (0.03)	ns
**CF**	Shape index end-diastolic rest (-)	0.66 (0.06)	0.67 (0.05)	ns
	Shape index end-diastolic stress (-)	0.71 (0.06)	0.72 (0.06)	ns
	Shape index end-systolic rest (-)	0.46 (0.09)	0.49 (0.07)	ns
	Shape index end-systolic stress (-)	0.51 (0.10)	0.55 (0.1)	0.03
	Eccentricity index rest (-)	0.88 (0.03)	0.88 (0.03)	ns
	Eccentricity index stress (-)	0.85 (0.04)	0.85 (0.03)	ns
**DDMC**	Shape index end-diastolic rest (-)	0.66 (0.06)	0.67 (0.05)	ns
	Shape index end-diastolic stress (-)	0.7 (0.07)	0.72 (0.06)	ns
	Shape index end-systolic rest (-)	0.45 (0.08)	0.49 (0.07)	ns
	Shape index end-systolic stress (-)	0.48 (0.10)	0.54 (0.09)	0.01
	Eccentricity index rest (-)	0.88 (0.03)	0.88 (0.03)	ns
	Eccentricity index stress (-)	0.85 (0.04)	0.85 (0.03)	ns
**DDMCCF**	Shape index end-diastolic rest (-)	0.66 (0.06)	0.68 (0.05)	ns
	Shape index end-diastolic stress (-)	0.71 (0.07)	0.72 (0.06)	ns
	Shape index end-systolic rest (-)	0.47 (0.09)	0.50 (0.06)	ns
	Shape index end-systolic stress (-)	0.51 (0.09)	0.56 (0.09)	0.02
	Eccentricity index rest (-)	0.88 (0.03)	0.88 (0.03)	ns
	Eccentricity index stress (-)	0.85 (0.04)	0.85 (0.03)	ns

**Table A3:** Mean LV functional variables compared between genders.

Parameter	Normal Perfusion Patients n = 39	Ischemic Patients n = 40	*p* Value
**End-diastolic rest (mL)**	104.9 [93.9, 115.9]	127.6 [116.8, 138.5]	0.004
**End-diastolic stress (mL)**	108.5 [96.9, 119.9]	138.0 [126.7, 149.4]	<0.001
**End-systolic rest (mL)**	33.0 [25.9, 40.0]	51.7 [44.8, 58.6]	<0.001
**End-systolic stress (mL)**	30.5 [23.1, 37.9]	57.5 [50.2, 65.8]	<0.001
**Left ventricular ejection fraction rest (%)**	69.4 [66.8, 72.0]	61.4 [58.8, 63.9]	<0.001
**Left ventricular ejection fraction stress (%)**	72.9 [70.3, 75.7]	60.1 [57.5, 62.7]	<0.001

**Table A4:** Mean LV geometrical variables compared between genders.

Parameter	Normal Perfusion Patients n = 39	Ischemic Patients n = 40	*p* Value
Shape index end-diastolic rest (-)	0.66 [0.64, 0.68]	0.67 [0.66, 0.69]	ns
Shape index end-diastolic stress (-)	0.69 [0.67, 0.71]	0.73 [0.71, 0.74]	0.007
Shape index end-systolic rest (-)	0.45 [0.43, 0.47]	0.49 [0.47, 0.52]	0.013
Shape index end-systolic stress (-)	0.47 [0.44, 0.50]	0.55 [0.53, 0.58]	<0.001
Eccentricity index rest (-)	0.88 [0.87, 0.89]	0.87 [0.86, 0.88]	ns
Eccentricity index stress (-)	0.86 [0.85, 0.87]	0.84 [0.83, 0.85]	<0.001
